# Understanding face identification through within-person variability in appearance: Introduction to a virtual special issue

**DOI:** 10.1177/1747021820959068

**Published:** 2020-09-28

**Authors:** Markus Bindemann, Graham J Hole

**Affiliations:** 1 School of Psychology, Keynes College, University of Kent, Canterbury, UK; 2 School of Psychology, University of Sussex, Brighton, UK

**Keywords:** Face, identification, matching, recognition, learning, within-person variability, multiple, natural, ambient, photographs, images

## Abstract

In the effort to determine the cognitive processes underlying the identification of faces, the dissimilarities between images of different people have long been studied. In contrast, the inherent variability between different images of the same face has either been treated as a nuisance variable that should be eliminated from psychological experiments or it has not been considered at all. Over the past decade, research efforts have increased substantially to demonstrate that this within-person variation is meaningful and can give insight into various processes of face identification, such as identity matching, face learning, and familiar face recognition. In this virtual special issue of the *Quarterly Journal of Experimental Psychology*, we explain the importance of within-person variability for face identification and bring together recent relevant articles published in the journal.

For many decades now, the study of face perception has been a mainstream topic in Psychology, investigated by scientists around the globe, with much of this research published in the *Quarterly Journal of Experimental Psychology*. One reason for this widespread interest is that the human face provides a highly accessible visual stimulus by which people can be identified and distinguished from one another. This identification process exists on a continuum. At one end of this continuum lies the identification of familiar people, who we know well, such as friends, colleagues, and family. We can typically accomplish identification of familiar faces with high accuracy, even under challenging conditions (see, for example, Bahrick et al., 1975; Lander et al., 2001; Morrison et al., 2000). At the other end of the continuum are the faces of unfamiliar people, who we have never met before. In contrast to familiar faces, circumstances that require identification of unfamiliar faces typically reveal substantial error rates (see, for example, Bruce et al., 1999, 2001; Kemp et al., 1997).

The identification of familiar and unfamiliar faces are both important in their own way. We need to identify familiar people to successfully navigate the diverse social settings of life, for example, to interact appropriately with the people closest to us, such as our immediate family, compared to other people, such as colleagues, who we also encounter routinely but in professional contexts. In contrast, identification of people who are unfamiliar to a viewer matters for important applied tasks, such as those requiring photo-ID checks for age verification in stores, access to restricted areas, person identification at airports and borders, and criminal investigations.

Both processes are typically also studied in different ways. The identification of familiar faces can be tested by asking viewers to recognise people that they know, based on their stored cognitive representations of these identities in memory. In its simplest form, recognition of familiar faces in psychological experiments can be tested by presenting a single photograph of a person and by asking participants to decide whether or not this person is familiar (Young et al., 1985), by asking them to name the person (Russell et al., 2009), or to classify faces based on stored semantic information such as occupation and nationality (Bindemann et al., 2005). In contrast, direct means to examine the identification of unfamiliar faces are visual matching tasks, in which observers have to decide whether two side-by-side faces depict the same person or different people (Burton et al., 2010; Fysh & Bindemann, 2018), or lineup tasks, in which a target has to be identified from among a set of faces (Bindemann et al., 2012; Bruce et al., 1999; Megreya & Burton, 2008).

These processes are clearly also linked, as every face that we know was once unfamiliar to us. Bridging the divide on the continuum between unfamiliar and familiar face identification is the process of face learning. In typical learning paradigms, observers are initially exposed to unfamiliar faces in photographs, videos, or during live interaction. The successful encoding and storage of such newly learned faces can then be tested directly, for example, with paradigms that require old or new decisions to photographs of faces (Bruce, 1982; Hill et al., 1997; Longmore et al., 2008), or indirectly, by looking for an advantage for learned over novel faces in tasks such as face matching (Bruce et al., 2001; Clutterbuck & Johnson, 2002, 2004; Megreya & Burton, 2006, 2007).

While familiar and unfamiliar face identification have now been researched extensively in Psychology, an important step-change has occurred in recent years in how these processes are studied. Traditionally, the study of face identification has relied on using controlled images of faces. For example, faces are often shown in a frontal view, with a neutral expression and under even lighting, and cropped to remove extraneous characteristics, such as hair and the image background. The rationale for such image manipulations is to remove “noise” from stimulus sets that might interfere with the controlled study of cognitive processes under laboratory conditions. Recently, however, researchers have begun to realise that this approach might limit the very tasks that were designed to understand face identification, by removing information that provides insight into how this task is accomplished in everyday life. This has given rise to a new research philosophy in which face identification is studied across images that depict the same person in different ways. For example, observers may be asked to compare pairs of face photographs that were captured under different conditions many months apart (Fysh & Bindemann, 2018; Megreya et al., 2013) or collected from relatively unconstrained image searches (Ritchie et al., 2015).

In some respects, this step-change in thinking in face research is similar to what happened long ago in memory research. For many decades following Ebbinghaus (1885/1913), the dominant approach to studying memory favoured the use of highly constrained stimuli, devoid of meaning and hence lacking any pre-existing associations for participants. This approach provided a great deal of useful knowledge about memory. However, significant progress was made from the 1960s onwards, once interest shifted to investigating how people actually remember real-world material in their everyday lives. Our awareness of the important role in memory of such phenomena as schemas, expectations, and inferences would not have been possible if the study of memory had continued to be confined to the study of memory for nonsense syllables.

Refocusing on face perception, the idea of studying identification with several images of a person is of course not new per se. Repetition priming paradigms, for example, have been in use for many decades. These experiments show that familiarity responses to known faces are faster at a test phase when these identities were also encountered during an earlier priming phase. These effects are typically measured across a change in image between prime and test phase to ensure that cognitive representations of face identity are engaged, rather than simple image-based processes (Bruce & Valentine, 1985; Burton et al., 1998; Ellis et al., 1987). Similarly, studies examining the identification of unfamiliar faces with eyewitness lineups and matching tasks have measured performance across different images of the same identities (Bruce et al., 1999; Henderson et al., 2001). In contrast to these types of studies, however, focus has recently shifted to more directly examine the *variability* that a person’s appearance exhibits in different photographs of their face, and how this can be used as an asset for understanding how faces are processed.

At the heart of this idea lies the observation that the many factors that are normally controlled in psychological experiments of face perception (e.g., viewpoint, lighting, expression) are present under natural viewing conditions and can interact in complex ways. The variability in appearance that these factors introduce is difficult to parameterise but is reflective of the conditions under which face identification typically has to be accomplished. Therefore, if we seek to understand how face identification operates in real life, it follows that this variation should be incorporated into psychological experiments. One method for achieving this is to use multiple *ambient* images of the same person. These are stimulus sets that are based on naturalistic pictures of faces, of the type that people normally have to recognise outside of the laboratory, such as those seen on social media, or images that reflect changes in facial appearance during interpersonal interaction. This approach presents a departure from how face identification has been studied in the past. Traditionally, research investigating face identification has focused on image differences between people. In this framework, the identity of a person is confounded with the specific face photograph at hand, and the task of face identification is conceptualised primarily as the visual differentiation of images. In turn, within-person variability is broadly considered as a nuisance variable that should be eliminated from psychological experiments.

Over the years, there have been occasions where the potential limitations of this approach have already been highlighted. Bruce (1994), for example, recognised the importance of within-person variability for understanding familiar face recognition, reasoning that experience of different photographs of a person is required for observers to gain insight into the aspects of a face that are stable across images. She proposed that establishing such “stability from variation” might reveal unchanging identity characteristics, while also providing an appreciation of the ways in which a face can vary. This could allow for boundaries to be established of what counts as possible instances of one person’s face, and not of another.

However, a compelling demonstration of these proposals did not arrive until years later, from research exploring whether the cognitive representations underpinning familiar face recognition might reflect image averages (Burton et al., 2005). These are statistical summaries that are derived by combining many different face images of the same person. In these averages, visual aspects that are not consistently present across different images of a person’s face are gradually eliminated, distilling a representation that summarises only shared identity information. In evaluations of this approach with different computational systems, an advantage for the recognition of face averages over individual face images has been found consistently (Burton et al., 2005; Jenkins & Burton, 2008, 2011; Robertson et al., 2015). This indicates that such statistical image summaries might present a good candidate for conceptualising representations of familiar faces that provide “stability from variation.”

As this line of research progressed, researchers were also able to demonstrate the importance of the variability that exists across different images of a person’s face. First, it became possible to characterise components of this variability during the averaging process, via the coefficients of principal component analysis of the contributing face images (Burton et al., 2011, 2016). This revealed that within-person variability in the appearance of a face is both *systematic*, so that key visual components of this variability can be isolated, but also *idiosyncratic*, whereby every person differs in how their facial appearance can vary.

In parallel, a powerful demonstration of the influence of within-person variability in facial appearance has emerged from some seemingly simple behavioural tasks. Jenkins et al. (2011) asked participants to sort 40 photographs of faces into piles, with each pile corresponding to a separate identity. The image set consisted of only two identities, with 20 images provided for each. These face images were unconstrained, to capture the variability that a person can exhibit naturally in appearance. Participants who were unfamiliar with the two identities sorted the images into a median of 7.5 piles (range = 3–16), with none of the 20 participants arriving at the correct answer. In marked contrast, participants who were familiar with the identities were almost perfect in their performance. This study demonstrates that, if you are unfamiliar with a person, there is a profound difficulty in grouping different images of their face together when these capture natural variability in appearance. Around the same time, evidence started to accumulate at an increasing rate to demonstrate identity confusions in face matching, whereby images of different people were also frequently mistaken as the same person (Bindemann et al., 2010; Burton et al., 2010; Megreya & Burton, 2006, 2008).

These studies demonstrate that within-person variability in facial appearance is substantial, to the point that observers often fail to realise that different photographs are of the same person. In turn, this within-person variability interacts with between-person similarity, increasing the possibility of coincidental resemblances between people, which are capable of generating many mistaken identifications. This challenges some common assumptions about faces, for example, that a (single) face image can be a reliable representation of a person’s identity or that face images are unique to each individual. Photo-identity documents, such as passports, are in ubiquitous international use on the basis of these assumptions. The psychological evidence suggests that the implementation of these identification methods, which currently do not capture within-person variability in appearance, is clouded in uncertainty.

This line of reasoning highlights the importance of incorporating within-person variability in appearance into experiments that investigate face identification. In this virtual special issue, we review a set of studies that have implemented this philosophy. We do not seek to provide a comprehensive review of this literature, which has grown rapidly in recent years (see, for example, Baker et al., 2017; Bindemann & Sandford, 2011; Kramer et al., 2016; Matthews & Mondloch, 2018; Murphy et al., 2015). Rather, our aim is to focus on recent articles published in this domain in the *Quarterly Journal of Experimental Psychology*. These papers reflect a growing appreciation of the importance of variability *as a source of information* that aids the process of face identification rather than hindering it.

The starting point for this virtual special issue is a theoretical paper (Burton, 2013) that drew together the key ideas presented here and outlined how an understanding of within-person variability in facial appearance could lead to much progress in this field. Burton (2013) specifically advocated the use of *multiple* images as a good way of building within-person variability in facial appearance into psychological experiments, so that identity is not confounded with individual photographs. He also suggested that if we seek to understand how faces are *actually* processed, then we should choose images that capture naturally occurring variation in appearance, under *ambient* viewing conditions, to reflect the type of conditions under which we have to identify people every day.

The other articles in this virtual special issue were published subsequently and have adopted some or all of these suggestions. Broadly, these articles fall into three categories, comprising research that has directly examined the benefit of studying face identification with multiple images (Andrews et al., 2015, 2017; Dowsett et al., 2016; Jones et al., 2017; Longmore et al., 2017; Menon et al., 2015; Ritchie & Burton, 2017); research with designs that incorporate multiple images of faces across *all* conditions (Bortolon et al., 2018; Hayward et al., 2017; Tuettenberg & Wiese, 2019); and recent work on voice recognition that is adapting these research methods from the face domain (Johnson et al., 2020; Lavan et al., 2019; Stevenage et al., 2020).

Several insights emerge from this collection of articles, which we summarise briefly here. First, these studies provide a coherent body of work to demonstrate that the provision of multiple photographs of a face improves identification accuracy. In one of the early studies, for example, Dowsett et al. (2016) asked participants to match photographs of a target to the correct identity presented within a set of 30 faces. Participants performed the task with just a single target photograph for identification, or with up to six different images. Identification accuracy improved consistently with the addition of each target photo, from 50% for a single image to around 90% when participants could draw on all six images. This demonstrates that, while a single photograph provides only limited information about a person’s identity, this problem can be overcome through insight into the variability in appearance that their face can exhibit. It is notable that these improvements did not generalise to other faces, consistent with the notion that within-person variability in facial appearance is idiosyncratic (Burton et al., 2011, 2016; Jenkins & Burton, 2011). Thus, learning how one face varies is limited in what it can reveal about variation in the appearance of other faces.

Other articles in this virtual special issue provide converging evidence that multiple images of the same face support improved identification and extend these findings in some important ways. Menon et al. (2015), for example, show that the provision of multiple images of a target only improves unfamiliar face matching if viewers are instructed to treat the images as the same identity, rather than as individual images of different people. Confirmatory evidence for this comes from Andrews et al. (2015), who asked observers to sort 40 face photographs into identities. In line with Jenkins et al.’s (2011) sorting task, this led to a substantial number of identification errors. Accuracy was virtually at ceiling, however, when participants were informed in advance that only two identities were present, akin to when this task is performed by observers who are familiar with the target identities (see Jenkins et al., 2011).

Andrews et al. (2015) also demonstrate that this sorting task provides a procedure for the incidental learning of face identities, which then improves performance for these targets in face matching tasks. Subsequent work indicates that this incidental learning technique also influences the N250 neural correlate of face familiarity, which suggests that image-independent face representations are beginning to be formed during sorting (Andrews et al., 2017). Taken together, the studies by Menon et al. (2015) and Andrews et al. (2015, 2017) therefore provide converging evidence that viewers are able to aggregate multiple images of the same face into abstractive identity representations, to become more *familiar* with a person, which in turn supports more accurate face matching.

In another paper in this virtual special issue, the importance of variability for face learning is clarified further, by shifting emphasis from the study of quantity to the quality of variation (Ritchie & Burton, 2017). This work demonstrates that high-variability image sets of the same face, which capture changes such as a person’s weight, age, hairstyle, and make-up, lead to better subsequent identification performance than low-variability image sets, in which such natural changes in a person’s appearance are not reflected. These effects are observed with the number of images held constant across low- and high-variability conditions, ruling out simple alternative explanations.

Two other studies in this virtual special issue provide insight into additional mechanisms by which multiple images of a face might enhance identification performance (Etchells et al., 2017; Longmore et al., 2017). These studies show that observers can use information from multiple images to improve identification of a face under previously unseen conditions. When a face is learned from a frontal *and* a profile view, for example, recognition of this person in a previously unseen mid-profile view is enhanced compared to when only one of the views was learned (Etchells et al., 2017). Similarly, learning images of a person’s face at 20 *and* 60 years of age leads to better recognition of that person from a novel image that falls between these ages (i.e., their 40-year-old face) compared to when the face had been learned from only one age-group image (Longmore et al., 2017). Thus, exposure to multiple photographs of the same face seems to allow for the interpolation of information, to facilitate identification of a person across a wider range of conditions.

The studies also hint at a potential avenue for artificially enhancing the benefit of multiple images for face identification. If observers can extract unseen views of faces that fall in between other available images, then it may also be possible to generate further views artificially to increase the number of images of a face that are available for identification, with some similar gains in performance. Jones et al. (2017) test this idea directly by employing a three-dimensional face-modelling technique to generate different views of a person from a single photograph. Learning faces from several of these synthesised views improved recognition compared to learning of a single front-view image. This effect was comparable to that obtained with corresponding sets of multiple photographs that captured the same face naturally. This raises the possibility that additional synthesised views can be computer-generated from a single face image to aid face learning and recognition when multiple photographs of a person are not available.

Overall, these studies demonstrate the importance of capturing within-person variability in facial appearance in psychological experiments, by revealing how the cognitive system exploits this variability for face learning and identification. In recognition of such findings, an increasing number of studies have now adopted the use of designs in which multiple images are provided for each person to further understanding in related areas of face perception. Hayward et al. (2017), for example, obtained photographs of faces from social media to capture identity across a range of natural appearances. These were used to study the learning of own- and other-race faces under conditions of greater ecological validity than could be achieved by previous studies that used only a single face image. Similarly, Tuettenberg and Wiese (2019) examined whether differences between own- and other-race faces during the identity-sorting of sets of photographs propagate to the subsequent matching and recognition of novel images of the learned identities. Bortolon et al. (2018) employed several ambient images per identity to study self-recognition under more natural viewing conditions than in previous studies. And the sorting tasks that have been developed to research within-person variability in face matching have now also been adapted to study vocal identity (Lavan et al., 2019; Stevenage et al., 2020) and to compare face and voice perception (Johnson et al., 2020). This demonstrates that the study of within-person variability continues to grow in interest and scope.

In conclusion, changes in the appearance of a person’s face across different photographs have long been treated as a nuisance variable in psychology experiments, whose influence should be eliminated prior to data collection. The alternative view, that such variability should be treated as meaningful because it can inform how faces are identified under natural viewing conditions, is reflected in the articles grouped together here. At the heart of this step-change in research practice is a simple truth that is often overlooked in the design of psychological experiments: how we study a cognitive process not only determines, but may also limit, what we can learn about it. Highly controlled experimentation has been of clear value for isolating key variables and processes that govern face perception. However, the simplification that has been inherent in such paradigms has also constrained knowledge gain, by removing the very information that enables face identification to proceed reliably in real life.^1^ The potential consequences of this approach are severe—if we conduct experiments that do not adequately capture the face processes that we seek to study, then we risk building models of these processes that are also limited in theoretical relevance (for an interesting discussion of this principle, see Burton et al., 2015). In turn, the studies presented here demonstrate that the integration of within-person variability into face learning and identification experiments can unlock substantial gains in accuracy. This offers promise also for devising better methods for applied settings that rely on such skills, such as personnel training in facial identification for police and border-control agencies (see, for example, Towler et al., 2019). The study of within-person variability now provides an exciting opportunity to address such gaps in our knowledge.

## Notes

1. There are parallels here with Marr’s (1982) account of how workers in early computer vision tried to simplify the problem of finding objects in a scene, by using a visually sparse “blocks world” of monochromatic geometric shapes—in the process unwittingly removing the very information (lawful and predictable spatiotemporal change) that facilitates object recognition in everyday life. Using this line of inquiry, any “success” in finding objects was generally confined to the blocks world environment. Marr argued that a superior approach (in the sense of one that produced solutions that could be generalised to real-world contexts) was to try to identify a computational goal plus a rigorous specification of the information by which this goal could be achieved. In the context of face recognition, the articles in this virtual special issue suggest that it might be fruitful to reconceptualise the nature of this information, treating variation in the input (the facial image) not as a nuisance or complication to be eliminated, but as *useful* information instead.
